# Exploring the Potential of DALL-E 2 in Pediatric Dermatology: A Critical Analysis

**DOI:** 10.7759/cureus.67752

**Published:** 2024-08-25

**Authors:** Subin Lim, Sarah Kooper-Johnson, Courtney A Chau, Sarah Robinson, Gabriela Cobos

**Affiliations:** 1 Dermatology, Eastern Virginia Medical School, Norfolk, USA; 2 Dermatology, Tufts Medical Center, Boston, USA; 3 Dermatology, Icahn School of Medicine at Mount Sinai, New York City, USA

**Keywords:** medical education, technological innovation, dermatology, pediatric dermatology, artificial intelligence

## Abstract

Introduction: Artificial intelligence (AI) is becoming increasingly explored for its potential applications in dermatology. Among various AI models, DALL-E 2 (San Francisco, CA: OpenAI), which generates de novoimages from textual inputs, has garnered significant attention for its remarkable photorealism. In our study, we aimed to analyze the performance of DALL-E 2 in the context of dermatology.

Methods: The following 12 pediatric dermatological conditions common to ages <15 years were selected as tests: acne, atopic dermatitis, contact dermatitis, vitiligo, congenital melanocytic nevus, warts, molluscum contagiosum, seborrheic dermatitis, alopecia areata, infantile hemangioma, impetigo, and dermatophytosis, specifically tinea corporis. Representative morphological descriptions of each diagnosis, along with their corresponding names, were inputted into DALL-E 2 as textual prompts and subsequently compared. The accuracy of the generated images and their alignment with the intended descriptions were assessed.

Results: Among the total of 24 images reported, 18 were photorealistic and six were cartoons. More cartoons were generated when providing the model with morphological descriptions as textual inputs compared to when diagnoses were inputted. While not entirely accurate, acne stood out as the only diagnosis that was the most consistent and closest to the actual diagnosis. Both images of acne portrayed erythematous papules scattered across the face. However, DALL-E 2 resulted in poor performance for the remaining eleven diagnoses. They did not accurately represent the intended diagnoses nor align with their counterpart image. Moreover, most of the generated images featured lighter skin tones.

Conclusion: In assessing DALL-E 2’s applications in dermatology, our study highlights the need for more domain-specific and demographically inclusive training data in its algorithms to improve performance.

## Introduction

Exploration of artificial intelligence (AI) for its potential applications in dermatology is rising. Our study aimed to analyze the dermatological performance of DALL-E 2 (San Francisco, CA: OpenAI), an intriguing AI model that generates photorealistic images based on textual prompts. The term “photorealistic” refers to a level of detail and accuracy that resembles a real, high-resolution photograph. Only one prior study has investigated its performance in dermatology by using diagnoses as textual inputs [1]. As such, our study aimed to further analyze DALL-E’s performance and its applications by utilizing characteristic morphological descriptions as textual inputs for common pediatric dermatological diagnoses [2,3].

## Materials and methods

DALL-E 2, an updated iteration of DALL-E, was utilized to generate all images. The standalone software with default settings was used. Compared to the original DALL-E, DALL-E 2 is known to generate more realistic and accurate images with four times greater resolution [4]. Utilizing DALL-E 2, images were generated for the following 12 pediatric dermatological conditions commonly seen in patients younger than 15 years old: acne, atopic dermatitis, contact dermatitis, vitiligo, congenital melanocytic nevus, warts, molluscum contagiosum, seborrheic dermatitis, alopecia areata, infantile hemangioma, impetigo, and dermatophytosis, specifically tinea corporis. For each condition, the following two textual prompts with a request to generate photorealistic images were inputted: a representative morphological description and the name of the diagnosis. The morphological descriptions were created based on classic descriptions that are often utilized in a clinical setting and were approved by a board-certified pediatric dermatologist. The complete prompts can be viewed in Table 1. Each input generated four images and the image that was deemed to be the most accurate by the majority vote among all authors of this manuscript was selected for analysis. Generated images from the first textual input were utilized, as DALL-E 2 generates different images with subsequent inputs of the same texts. These images were evaluated for overall accuracy, concordance between outputs for the two types of textual inputs, and whether they appeared photorealistic or cartoonish. In our study, photorealistic images were defined as portraying realistic photos of humans, whereas cartoon representations featured simplified visual elements and less realistic portrayals of human features. All images presented in the figures are AI-generated and do not depict real individuals. No real patient data or images were used as reference or inspiration for the AI-generated images.

**Table 1:** Textual prompts of morphological descriptions and diagnoses used for generating images of common pediatric dermatological conditions in DALL-E 2. DALL-E 2 (San Francisco, CA: OpenAI)

Diagnosis	Morphological description input	Diagnosis input
Acne vulgaris	Please generate a photorealistic image of a child with papules and pustules distributed over the forehead, cheeks, and nose with comedones.	Please generate a photorealistic image of a child with acne vulgaris on the face.
Atopic dermatitis	Please generate a photorealistic image of a child with pruritic, pink, red papules and vesicles with exudation and crusting on bilateral cheeks of the face.	Please generate a photorealistic image of a child with atopic dermatitis on the bilateral cheeks of the face.
Contact dermatitis	Please generate a photorealistic image of a child with itchy, erythematous, scaly patches, and plaques on the thighs.	Please generate a photorealistic image of a child with contact dermatitis on the thighs.
Vitiligo	Please generate a photorealistic image of a child with asymptomatic, depigmented macules and patches on the hands.	Please generate a photorealistic image of a child with vitiligo on the hands.
Congenital melanocytic nevus	Please generate a photorealistic image of a child with a large, irregular-bordered, brown plaque with hypertrichosis within the lesion on the back.	Please generate a photorealistic image of a child with a congenital melanocytic nevus on the back.
Wart	Please generate a photorealistic image of a child with a well-circumscribed, dome or cauliflower-shaped, grayish brown, hyperkeratotic skin growth on the foot.	Please generate a photorealistic image of a child with a wart on the foot.
Molluscum contagiosum	Please generate a photorealistic image of a child with pearly, white to pink, dome-shaped, umbilicated 3 mm papules on the arm.	Please generate a photorealistic image of a child with molluscum contagiosum on the arm.
Seborrheic dermatitis	Please generate a photorealistic image of a child with scaly, flaky, erythematous, greasy, thick white plaques on the scalp.	Please generate a photorealistic image of a child with seborrheic dermatitis on the scalp.
Alopecia areata	Please generate a photorealistic image of a child with round, sharply demarcated, unscarred, hairless patches on the scalp.	Please generate a photorealistic image of a child with alopecia areata on the scalp.
Infantile hemangioma	Please generate a photorealistic image of a child with a bright, red tumor in the superficial dermis with a lobulated, irregular surface.	Please generate a photorealistic image of a child with an infantile hemangioma on the arm.
Impetigo	Please generate a photorealistic image of a child with inflamed pustules and honey-colored crusts around the mouth.	Please generate a photorealistic image of a child with impetigo around the mouth.
Dermatophytosis (tinea corporis)	Please generate a photorealistic image of a child with annular lesions with scaly raised borders and central clearing on the arm.	Please generate a photorealistic image of a child with tinea corporis on the arm.

## Results

**Figure 1:**
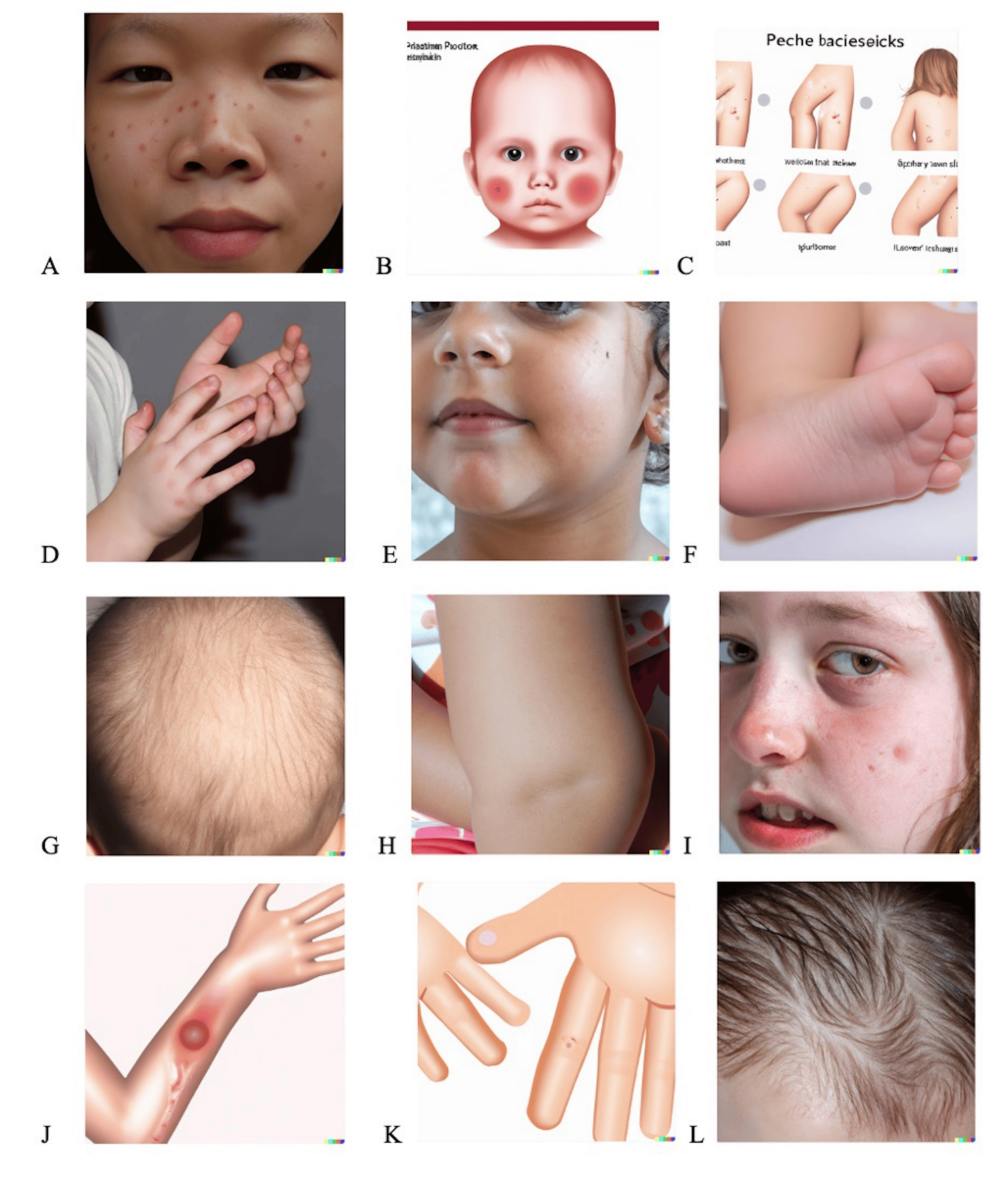
Output of text-to-image generation by DALL-E 2 in response to input describing the morphological description of common pediatric dermatological diagnoses. The images show (A) acne vulgaris, (B) atopic dermatitis, (C) contact dermatitis, (D) vitiligo, (E) congenital melanocytic nevus, (F) wart, (G) alopecia areata, (H) infantile hemangioma, (I) impetigo, (J) dermatophytosis (tinea corporis), (K) molluscum contagiosum, and (L) seborrheic dermatitis. DALL-E 2 (San Francisco, CA: OpenAI) All images are AI-generated and do not depict real individuals.

**Figure 2:**
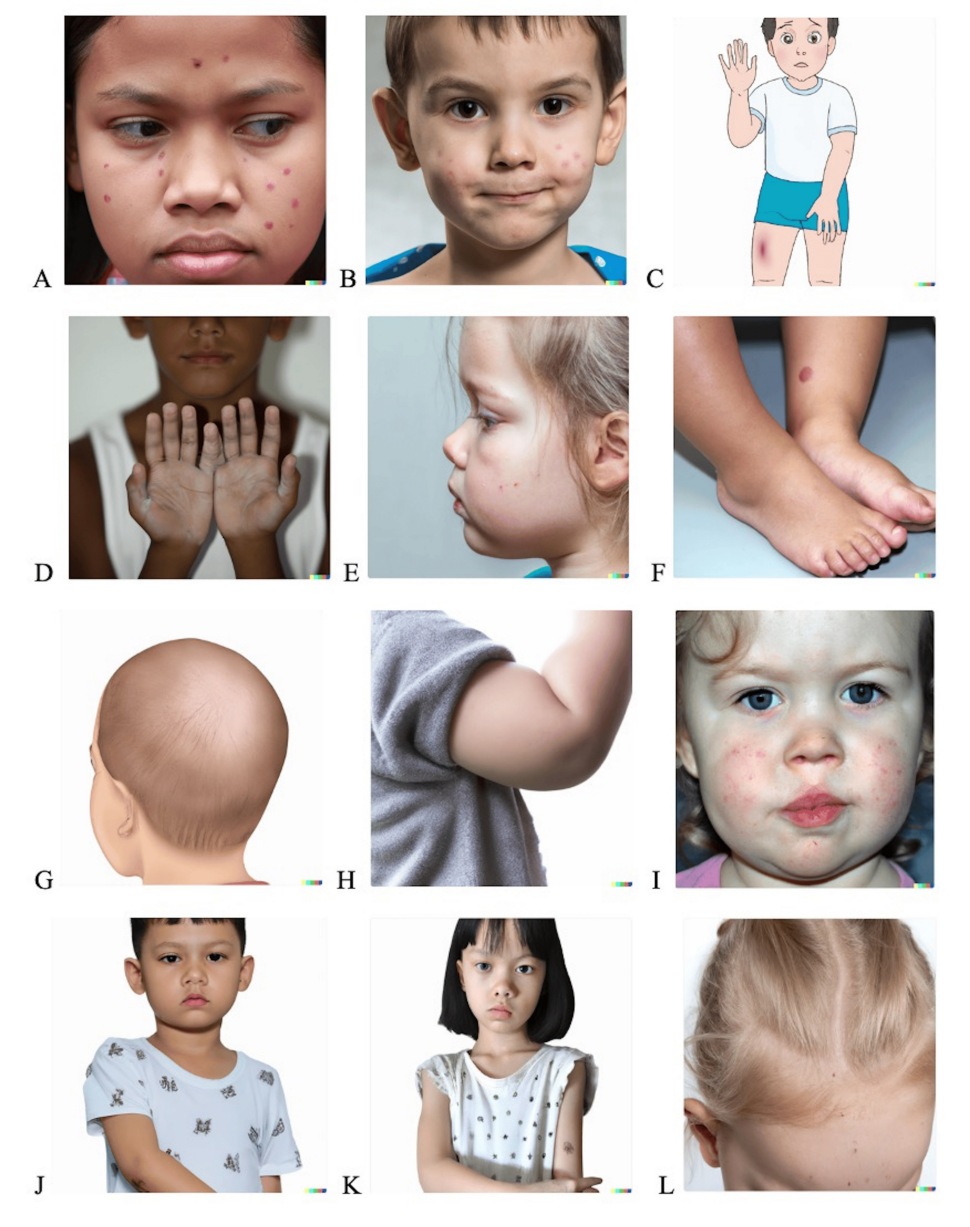
Output of text-to-image generation by DALL-E 2 in response to inputting the diagnosis name of common pediatric dermatological diagnoses. The images show (A) acne vulgaris, (B) atopic dermatitis, (C) contact dermatitis, (D) vitiligo, (E) congenital melanocytic nevus, (F) wart, (G) alopecia areata, (H) infantile hemangioma, (I) impetigo, (J) dermatophytosis (tinea corporis), (K) molluscum contagiosum, and (L) seborrheic dermatitis. DALL-E 2 (San Francisco, CA: OpenAI) All images are AI-generated and do not depict real individuals.

As seen in Table 1, both the morphological description and the name of the diagnosis of the pediatric dermatological conditions were inputted into DALL-E 2 to generate images. A total of 24 images were assessed with two images representing each diagnosis (Figures 1A-1L and Figures 2A-2L). Eighteen images were photorealistic (Figures 1A, 1D-1I, 1L and Figures 2A, 2B, 2D-2F, 2H-2L), and six were cartoon representations (Figures 1B, 1C, 1J, 1K and Figures 2C, 2G). More cartoons were generated with morphological descriptions as inputs (n=4, 16.7%) compared to when diagnoses were used (n=2, 8.3%).

Among all generated images, acne was the only diagnosis for which images were mostly consistent and accurate. Both images, one generated with a morphological description input (Figure 1A) and the other with a diagnosis input (Figure 2A), portrayed erythematous papules scattered across the face. However, other common features of acne, such as comedones, pustules, post-inflammatory hyperpigmentation, or evidence of background scarring, were not appreciated. For the remaining 11 diagnoses, DALL-E 2 performed poorly. For instance, 25% (6/24) of images were cartoon representations of the conditions, and they all failed to accurately reflect their respective medical diagnoses. Among the cartoon representations, alopecia areata was the only diagnosis that seemed to most closely reflect the actual condition. In this case, the model created diffuse areas of hair loss around the vertex instead of discrete patches, which is more pathognomonic for alopecia areata.

Among the photorealistic images generated, atopic dermatitis (Figure 2B), seborrheic dermatitis (Figure 1L), and impetigo (Figure 2I), were the only dermatologic conditions that retained some morphological features commonly seen in these diagnoses. For example, for atopic dermatitis, although DALL-E 2 did not produce an image with “pruritic, pink, red papules and vesicles” on the face of a child, the AI created an image with multiple ill-defined, pink patches on the face, which could be observed in pediatric patients with atopic dermatitis. Additionally, while the image for seborrheic dermatitis fell short in capturing the white scales and plaques typically associated with the condition, the model did effectively simulate a greasy-appearing scalp. Lastly, for impetigo, while there was no evidence of honey-yellow crusting indicating bacterial infection, the photorealistic image of impetigo portrayed erythematous plaques and a few excoriated papules, which may be seen with this condition. For the remaining diagnoses - contact dermatitis (Figures 1C, 2C), vitiligo (Figures 1D, 2D), congenital melanocytic nevus (Figures 1E, 2E), wart (Figures 1F, 2F), infantile hemangioma (Figures 1H, 2H), and molluscum contagiosum (Figures 1K, 2K) - no clinical features known to these conditions could be adequately appreciated in the respective AI-generated images.

Nevertheless, most of the generated images for each diagnosis were inconsistent between the two textual prompts, and evaluating consistency was challenging due to the significant inaccuracies depicted in the images. Moreover, notably, most images featured lighter skin tones (Figures 1A-1L and Figures 2A-2L).

## Discussion

As observed in our study, acne was the only diagnosis for which images were mostly consistent and accurate. Likewise, a previous study assessing the dermatologic domain knowledge of DALL-E 2 identified acne as the test case with the highest accuracy [1]. The overall inaccuracy of the generated images is likely attributable to a flaw in the training data rather than the software itself. Currently, DALL-E 2 relies on non-specific training data, which limits the model from learning the nuanced features of medical diagnoses, leading to less accurate depictions of diseases. To enhance the accuracy of dermatological image generation, training DALL-E 2 on dermatological-specific images and descriptions could be effective in capturing the intricacies of various diagnoses. For example, a prior study explored the results of fine-tuning Guided Language to Image Diffusion for Generation and Editing (GLIDE), another text-to-image generative AI, to see if it could be refined for dermatological applications [5]. As hypothesized above, this study demonstrated the successful fine-tuning of GLIDE on 10,015 dermoscopic images to generate synthetic dermoscopic images. Although the generated images revealed varying degrees of quality and realism as our study did, with melanocytic nevi and melanoma being the most accurate, the AI assessment showed superior classification performance compared to the dermatologist [5]. As a result, through the incorporation of diverse data sets and domain-specific knowledge, AI models, especially text-to-image generators, hold a promising potential for various clinical and educational uses in dermatology.

With the advancement of AI, there continues to be an increasing number of studies evaluating the use of AI in various medical settings [1,4,5]. A study assessing DALL-E 2’s knowledge in radiology revealed that while it was able to generate x-ray images with fairly accurate anatomical proportions seen in real x-rays, it fell short when trying to convey pathology, such as images of fractures and tumors [6]. As such, DALL-E 2 performed poorly in generating computed tomography (CT), magnetic resonance imaging (MRI), and ultrasound images [6]. Given that both radiology and dermatology are fields that heavily rely on visual detection of pathology, this study corroborates our findings with regard to the need for fine-tuning these AI models to relevant medical data and terminology to improve their performance in medical settings. 

Darker skin types were also starkly underrepresented in our study, indicating a deficiency in diversity within the training data. This aligns with the findings of a prior study that revealed the ongoing challenges of AI when evaluating dermatologic conditions in skin of color (SOC) patient populations [7]. Only 30% of the artificial intelligence programs evaluated in this study had reported their use in dermatology, specifically SOC. Again, this was largely due to the largely underrepresented SOC images in AI training data sets [7]. As highlighted by Guo et al., there is a need for dermatologic AI and machine learning studies to incorporate more diverse and demographically inclusive datasets into their algorithms to ensure improved performances across all patient groups [8]. Considering the current disparity among dermatological educational resources, it is crucial that training datasets for any algorithm represent all demographics, especially skin of color [9]. This is particularly relevant for the pediatric population due to the inherently diverse nature of skin conditions within this cohort. Other limitations of this study include the use of a single image generator and the lack of available standardized tools to assess image quality.

Nevertheless, the integration of AI holds promise for improving clinical accuracy in dermatology. However, with the increasing implementation of AI in dermatology, there are significant ethical considerations. Given the potential for inaccuracy in AI-generated images, one major concern is possible misdiagnosis. Additionally, the use of AI in generating medical imagery could perpetuate stereotypes, particularly if the training data is biased. Making incorrect clinical assessments and reinforcing pre-existing biases have the potential to impair the quality of care provided to diverse patient populations. Other ethical implications are related to autonomy, informed consent, and privacy. Clarifying the role of AI, ensuring transparent communication between patients and AI developers, and acknowledging the limitations of AI-generated images would be crucial to mitigate these risks [10]. Ensuring to collaboratively develop and meet regulatory guidelines for the use of AI would be essential to ethically and effectively incorporate AI in dermatology.

## Conclusions

Our study demonstrated that while DALL-E 2 could generate photorealistic images for some common pediatric dermatological conditions, such as acne, it often produced inaccurate and inconsistent depictions of most other pediatric dermatological diagnoses. This is likely due to non-specific training data not tailored to dermatological information, resulting in mostly cartoon representations and a notable underrepresentation of diverse skin tones. As such, there is a strong need for fine-tuning AI models with dermatological-specific and demographically inclusive training data in order to improve the accuracy, reliability, and clinical utility of DALL-E 2 in dermatology. With these modifications in the future, DALL-E 2 could enhance dermatological trainee and patient education and support medical practice through accurate visual references that may assist in diagnosis and treatment planning. Nevertheless, ethical implications of using AI-generated images, such as the potential for misdiagnoses, perpetuation of biases, autonomy, and informed consent, must be rigorously assessed to ensure equity and safeguarding of patient privacy.

## References

[1] Cheraghlou S (2023). Evaluating dermatologic domain knowledge in DALL-E 2 and potential applications for dermatology-specific algorithms. Int J Dermatol.

[2] (2023). How DALL-E 2 actually works. https://assemblyai.com/blog/how-dall-e-2-actually-works/.

[3] Ramesh A, Pavlov M, Goh G (2012). Zero-shot text-to-image generation. arXiv.

[4] Dall-e 2 DALL·E 2. https://openai.com/index/dall-e-2/.

[5] Shavlokhova V, Vollmer A, Zouboulis CC (2023). Finetuning of GLIDE stable diffusion model for AI-based text-conditional image synthesis of dermoscopic images. Front Med (Lausanne).

[6] Adams LC, Busch F, Truhn D, Makowski MR, Aerts HJ, Bressem KK (2023). What does DALL-E 2 know about radiology?. J Med Internet Res.

[7] Fliorent R, Fardman B, Podwojniak A (2024). Artificial intelligence in dermatology: advancements and challenges in skin of color. Int J Dermatol.

[8] Gordon ER, Trager MH, Kontos D, Weng C, Geskin LJ, Dugdale LS, Samie FH (2024). Ethical considerations for artificial intelligence in dermatology: a scoping review. Br J Dermatol.

[9] Guo LN, Lee MS, Kassamali B, Mita C, Nambudiri VE (2022). Bias in, bias out: underreporting and underrepresentation of diverse skin types in machine learning research for skin cancer detection - a scoping review. J Am Acad Dermatol.

[10] Ebede T, Papier A (2006). Disparities in dermatology educational resources. J Am Acad Dermatol.

